# Special Issue: Cellular Oxygen Homeostasis

**DOI:** 10.3390/ijms23094505

**Published:** 2022-04-19

**Authors:** Verena Tretter

**Affiliations:** Department of Anesthesia, General Intensive Care and Pain Management, Medical University of Vienna, 1090 Vienna, Austria; verena.tretter@meduniwien.ac.at

Oxidative phosphorylation is an efficient way to generate the cellular energy currency ATP in a cascade of redox reactions, which ultimately terminate in the reduction of molecular oxygen to water. Partial reduction of oxygen yields intermediate radical or non-radical oxygen species (ROS), that, similar to other reactive species, such as the evolutionary older reactive sulfur species (RSS), have high reactivity towards biomolecules and can change their structure and function. ROS at low concentrations have signaling functions, but higher amounts can have detrimental consequences for the viability of a cell. Interestingly, both excess and deficiency of oxygen can induce oxidative distress, therefore, maintaining oxygen homeostasis is an important factor to ensure proper cellular function. Different tissues in the human body are physiologically exposed to different partial pressures of oxygen, therefore “normoxia” needs to be seen in a cell type-specific context, which is defined as providing an optimal condition for the physiology of this special cell or tissue. In a certain range, cells have the capability to adapt to altered oxygen conditions (=relative hypoxia or hyperoxia) by activating transcriptional programs that adjust metabolic pathways according to the availability of substrates. They can also increase the antioxidative defense and stress pathways in order to fight off excessive damage by reactive species.

This Special Issue, “Cellular Oxygen Homeostasis”, presents a collection of articles (five original articles and three reviews) that investigate different aspects of cellular oxygen homeostasis or discuss the current understanding.

Fratantonio et al. [1] investigated molecular mechanisms related to the normobaric oxygen paradox (NOP) in peripheral blood mononuclear cells (PBMCs). They show that, depending on the intensity of the hyperoxic stimulus, cells stimulate different transcriptional responses upon return to normoxia. Hypoxia inducible factor-1α (HIF-1α) is activated after mild hyperoxia (NOP). However, after stronger hyperoxic episodes, increased stress response is mediated by nuclear factor kappa-light-chain-enhancer of activated B cells (NF-KB).

Ivanova and Lyublinskaya [2] discuss redox homeostasis in pluripotent stem cells. Originating from the hypoxic blastocyst of the uterus, these stem cells have special features, such as high DNA damage repair and hindered senescence due to telomerase activity. Their metabolism shows significant plasticity in order to adjust redox metabolism to the microenvironment as well as to the different states of pluripotency. Generally, a higher share of anaerobic glycolysis is used to generate cellular energy. Their self-renewal process in proliferation, as well as their capability to differentiate into different cell types of the three germ layers, is regulated by ROS and, as such, is tightly regulated.

Mochizuki-Kashio et al. [3] take a closer look at the hematopoietic stem cell (HSC) fate and the importance of mitochondrial and lysosomal organelle homeostasis. Quiescent HSC are located in the hypoxic bone marrow until they start to proliferate and differentiate in order to supply hematopoietic cells. Glycolysis and oxidative phosphorylation are used to a variable degree depending on the hematopoietic cell type. Dynamics of mitochondria (fission/fusion/mitophagy), as well as of lysosomes adjust organelle volume according to the stem cell potential. The role of the tumor suppressor folliculin as important regulator of these processes is discussed in further detail.

Hypoxia is also an important factor in many tumors and induces metabolic adaptations making the tumor more aggressive and therapy resistant. Many of the cell responses to hypoxia are orchestrated by the transcription factor HIF-1, that regulates more than 1000 genes. Ziolkowska-Suchanek et al. [4] identified the HIF-1α induced Rho-GAP protein FAM13A upregulated under hypoxia in non-small cell lung cancer cells (NSCLC), and show its importance in proliferation, cell cycle regulation, cell migration and metastasis.

ROS-mediated cytotoxicity is used to treat cancer by photodynamic therapy (PDT). A photosensitizer accumulates in cancer tissue and is excited with light of a suitable wavelength in order to generate ROS. A common side effect is photosensitive disorder, which can be ameliorated by using novel sensitizers, such as porphylipoprotein (PLP). Kurokawa et al. [5] investigated the efficacy of PLP PDT with regard to uptake into cholangiocarcinoma cells, identify heme carrier protein 1 (HCP1) as a putative uptake transporter and show evidence, that PLP PDT can inhibit tumor growth in mice.

Some pathological states or diseases, such as hyperglycemia and diabetes induce a state of pseudohypoxia due to a decreased NAD+/NADH ratio. Owczarek et al. [6] show that the transcription factor carbohydrate response element binding protein (ChREBP) is an important transcriptional regulator of HIF-1α expression in epithelial cells of renal proximal tubules in response to high glucose levels.

The gut is an interesting model system of oxygen sensitivity, as it contains variable degrees of hypoxia at its epithelial barrier. Homeostasis is maintained by an intact microbiome-gut communication, that regulates nutrient absorption, barrier function and immune responses. Konjar et al. [7] review the important aspects of oxygen and microbiota, the sensitive balance of metabolites and detrimental developments leading to inflammation.

It is necessary to understand that cellular redox homeostasis relies on a complex interdependent network of pro- and anti-oxidants and redox coupling. In many cases in the context of diseases and therapy it is difficult to discern and to decipher relationships of cause and effects. This might be one reason why antioxidative therapies have repeatedly turned out to not be successful, as a non-specific one-size-fits-all approach does not consider the necessary specificity, dosage, and targeting of the drug. In the review article by our group [8] we give an overview of the complex systems of redox regulation, reactive species and the cellular antioxidative defense and discuss their importance in various pathological developments. We hypothesize that diseases exhibiting oxidative stress may benefit from a precision medicine approach to provide better treatment for patients.
